# Simulation Study of Different OPM-MEG Measurement Components

**DOI:** 10.3390/s22093184

**Published:** 2022-04-21

**Authors:** Urban Marhl, Tilmann Sander, Vojko Jazbinšek

**Affiliations:** 1 Faculty of Natural Sciences and Mathematics, University of Maribor, Koroška cesta 160, 2000 Maribor, Slovenia; 2 Department of Physics, Institute of Mathematics, Physics and Mechanics, Jadranska ulica 19, 1000 Ljubljana, Slovenia; vojko.jazbinsek@imfm.si; 3 Physikalisch-Technische Bundesanstalt, Abbestraße 2, 10587 Berlin, Germany; tilmann.sander-thoemmes@ptb.de

**Keywords:** magnetoencephalography, optically pumped magnetometers, superconducting quantum interference device, volume conductor, boundary element method, equivalent current dipole, source localization, ambient noise, spontaneous brain noise

## Abstract

Magnetoencephalography (MEG) is a neuroimaging technique that measures the magnetic fields of the brain outside of the head. In the past, the most suitable magnetometer for MEG was the superconducting quantum interference device (SQUID), but in recent years, a new type has also been used, the optically pumped magnetometer (OPM). OPMs can be configured to measure multiple directions of magnetic field simultaneously. This work explored whether combining multiple directions of the magnetic field lowers the source localization error of brain sources under various conditions of noise. We simulated dipolar-like sources for multiple configurations of both SQUID- and OPM-MEG systems. To test the performance of a given layout, we calculated the average signal-to-noise ratio and the root mean square of the simulated magnetic field; furthermore, we evaluated the performance of the dipole fit. The results showed that the field direction normal to the scalp yields a higher signal-to-noise ratio and that ambient noise has a much lower impact on its localization error; therefore, this is the optimal choice for source localization when only one direction of magnetic field can be measured. For a low number of OPMs, combining multiple field directions greatly improves the source localization results. Lastly, we showed that MEG sensors that can be placed closer to the brain are more suitable for localizing deeper sources.

## 1. Introduction

Magnetoencephalography (MEG) is a non-invasive imaging technique in neuroscience which determines activity within the cortex on the basis of measurements of the magnetic field near the head [1]. It has very good temporal and fair spatial resolution. To achieve a high spatial resolution, a sophisticated source reconstruction method has to be used, which considers the geometry of each subject’s head [2] obtained from reconstructed magnetic resonance images (MRI). The main disadvantage of the standard MEG system is the requirement for liquid helium to cool the superconducting SQUID magnetometers. In the last decade, commercial optically pumped magnetometers (OPM) based on alkaline metal vapor have appeared on the market, which have up to five times lower sensitivity than the standard SQUID magnetometers but they do not need cooling [3,4]. They are fully suitable for brain magnetic field measurements [5].

The availability of a new type of sensor triggered the evaluation of source analysis methods for OPM-MEG [5,6,7] derived from methods developed for SQUID-MEG. However, MEG systems with OPM sensors are still under development; therefore, commercial complete MEG systems are rare, and the current experimental systems all have different configurations [6,8,9,10], complicating the evaluation of analysis methods.

The magnetic fields produced by the brain currents accompanying neural activity are very weak. Since the OPM sensors do not need cooling with cryogenics, they can be placed closer to the brain. The latest theoretical and experimental research results show that this significantly increases the measured magnetic signal and thus the signal-to-noise ratio (SNR) [6,7,8]. Currently, there are commercial magnetometers on the market which can simultaneously measure more than one orthogonal direction of the magnetic field. One example is the magnetometer QZFM Gen-3, made by QuSpin (https://quspin.com/qzfm-gen-3/, accessed on 6 April 2022), which can measure all three orthogonal components of the magnetic fields [4]. The operating principle of the triaxial OPM sensor was nicely explained in a recent study by Boto et al. [11]. An interesting question is which component is the most suitable for extracting physiologically relevant information from MEG. A simulation study by Iivanainen et al. [6] showed that the highest field amplitude is obtained by the OPM sensor that measures the normal component. They also showed that using two or three components of the sensor increases the capacity of the captured information.

A recent theoretical study of a triaxial OPM system by Brookes et al. [12] showed that using all three sensor components increased the ability to differentiate actual brain activity from sources of magnetic interference when using the beamforming source localization approach. Additionally, they showed that a triaxial OPM-MEG system is less prone to artifacts due to head movements in the magnetic fields. Although studies have been carried out on the impact of noise on OPM-MEG measurements, there are still many open research questions. MEG measurements are carried out in a variety of environments. The magnetically shielded rooms vary considerably between research groups, and so does the noise intensity. Therefore, how the MEG systems (OPM or SQUID) with different measurement components behave for varying noise levels is an important question. To explore this, we simulated current dipoles inside a brain for multiple sensor components of both OPM- and SQUID-MEG. We added two types of noises (spontaneous brain noise and ambient noise) to the simulated magnetic fields. We tested how their strength affected the localization error and SNR. Our study differs from [12] because here, we used a more sophisticated forward model and a different source localization method.

Among the most established approaches used to place individual OPM sensors on the head is the method of 3D printing a custom sensor holder, which fits the individual subject’s head surface [8,13]. This allows us to place sensors easily in various locations around the scalp as required by the user’s specific neuroscience question. Many research groups are currently limited by the number of OPM sensors. One of the reasons for this is the cost of several thousand dollars per sensor. Therefore, we explored how lowering the sensor count affected the source localization results of an equivalent current dipole (ECD) fit. The most important question is whether combining multiple components lowers the source localization error in the case of a lower sensor count. This has partly been explored already in previous works. Matsuba et al. [14] calculated dipole localization errors in relation to the number of sensors for normal and vector SQUID magnetometers. They showed that for high sensor counts, the localization error is independent of the number of sensors. These results do not directly apply to OPM sensors, which are much closer to the head. The authors of [12] concluded that 50 triaxial sensors have an approximately similar performance to 80 radial sensors in the absence of interference. An interesting question remains: how does an OPM-MEG system with a triaxial sensor configuration compare with dual- or single-sensor configurations for counts lower than 50? We hypothesize that the localization error for low sensor counts will be much lower for the triaxial configuration compared with a single-sensor configuration.

Although it is possible to measure deep brain sources with classical SQUID-based MEG [15,16,17], one of the proposed advantages of on-scalp MEG is the ability to measure it more accurately [18,19]. This was briefly explored in [7], where they concluded that OPMs offer better coverage of deep brain areas due to improved SNR. How the performance of different MEG systems (OPM and SQUID) varies for different depths is still unexplored. In our work, we systematically explored how signal strengths and localization errors changed for different sensor configurations for varying dipole depths. We are particularly interested in whether combining multiple components of the OPM-MEG systems offers any advantages.

In this study, we analyzed the performance of three orthogonal measurement components (one normal to the head surface, and two tangential along the lines of latitude and longitude) of two types of MEG systems (OPM-MEG and SQUID-MEG). We carried out a simulation of many single sources inside the brain with added noise. We incorporated two types of noises: spontaneous brain activity [7] and random ambient noise. Preliminary results [20] showed that the normal component had the highest SNR when spontaneous brain noise was added to the simulated fields. Here, we checked how the strength of different noises affects the localization error of dipole fit (the distance between two dipoles, relative error, and correlation coefficient) [21] and the SNR. Next, we checked how lowering the sensor count affected the source localization error for different OPM-MEG configurations. Lastly, we performed simulations for sources at different depths inside the head. Although the simulations were made with OPM sensors as an example, the results are relevant for any other MEG sensor which can be placed close to the subject’s scalp, e.g., high-temperature SQUIDs [22].

## 2. Materials and Methods

**Figure 1:**
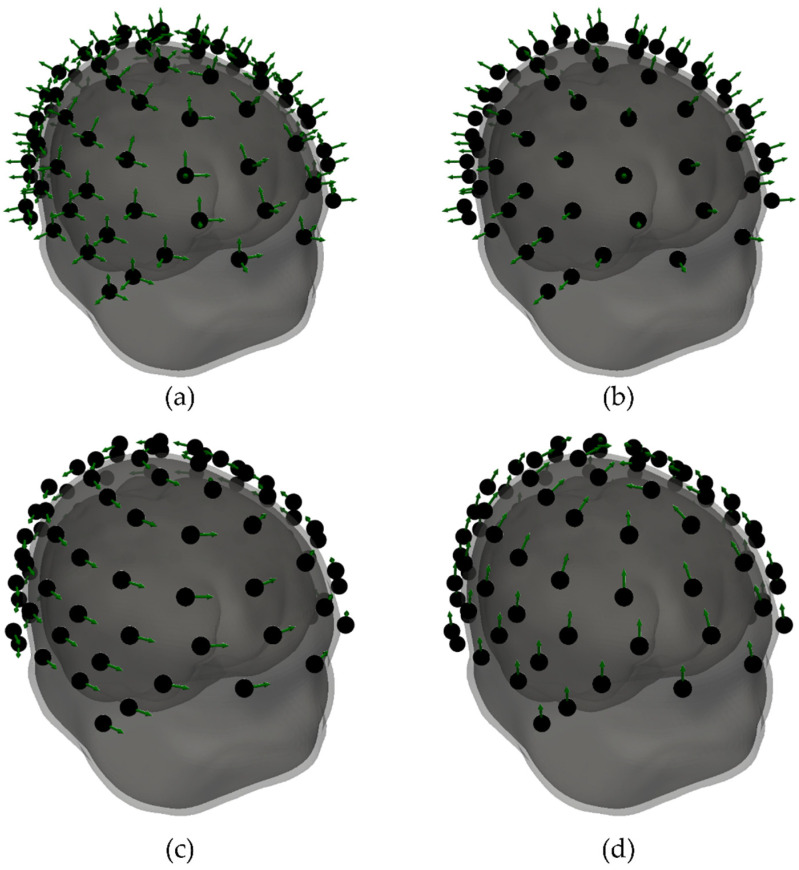
Different configurations of OPM-MEG for one selected subject: (**b**) normal components (OPM-NOR), (**c**) tangential components along lines of latitude (OPM-TAN-LAT), (**d**) longitude (OPM-TAN-LON) and (**a**) all three components combined (OPM-ALL). The gray surfaces show individual BEM mesh model layers. The center of the black dots represents the center of the OPM sensors, and the green arrows show the sensors’ sensing directions. The sensor count for this subject is 93.

In the simulations, we calculated magnetic field maps for two systems with different types of sensors: the OPM-MEG system and the SQUID-MEG system. For the OPM-MEG system, the calculations were made with 4 different combinations of sensor orientations: normal components relative to the subject’s scalp only (OPM-NOR), tangential components along the lines of latitude relative to the subject’s scalp or oriented horizontally only (OPM-TAN-LAT), tangential components along the lines of longitude or oriented “vertically” only (OPM-TAN-LON), and using all three orthogonal components simultaneously (OPM-ALL). The OPM sensors were distributed around the head for each subject separately. The number of sensors was limited by the physical size of typical commercial sensors, with a footprint in the range of 10 × 15 mm; therefore, we placed more sensors on subjects with bigger heads. On average, we placed 82 sensors; the highest sensor count was 93 and the lowest was 71 sensors. For one subject, we show the sensors for all four configurations (Figure 1). When performing the simulation, we took the geometry of the QuSpin QZFM Gen 2.0 sensors into account [4]. The magnetic field was calculated by integrating over 8 points inside the sensor (the corners of the cube) with the same weights.

**Figure 2:**
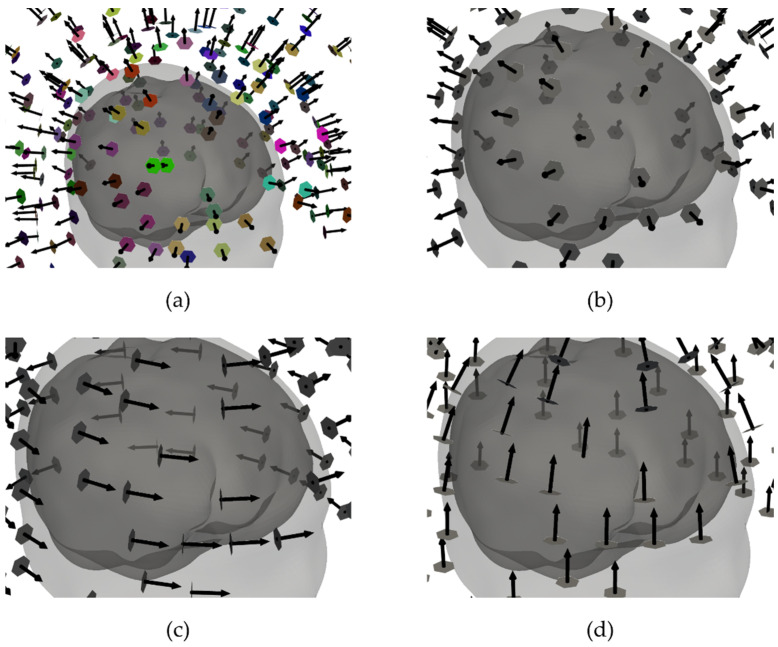
Different configurations of the SQUID measurement components for one selected subject: (**a**) the original Yokogawa system with 125 axial gradiometers magnetometers (SQUID-AXI-GRAD), (**b**) normal magnetometers (SQUID-NOR), (**c**) tangential magnetometers along the lines of latitude (SQUID-TAN-LAT) and (**d**) longitude (SQUID-TAN-LON). In the gradiometer system, the individual sensor pairs which form a gradiometer have the same color.

For the SQUID-MEG system, we tested 4 different configurations. The geometry of the SQUID-MEG system was based on a commercial SQUID system with 125 first-order axial gradiometers, produced by the company Yokogawa (Tokyo, Japan) [23] (SQUID-AXI-GRAD). Since the OPM-MEG configurations use magnetometers only, we created SQUID layouts with three orthogonal magnetometers located close to the head from the gradiometer pair in SQUID-AXI-GRAD: normal components (SQUID-NOR), and tangential components along the lines of latitude (SQUID-TAN-LAT) and along the lines of longitude (SQUID-TAN-LON). All SQUID-MEG layouts are shown in Figure 2. For the SQUID-AXI-GRAD layout, we took the real sensor geometry of the Yokogawa MEG system into account [23]. The magnetic field was calculated by integrating over 4 points for each pickup coil of the gradiometer. For the magnetometer layouts, the calculation was the same, but we integrated over only one pickup coil. 

**Figure 3:**
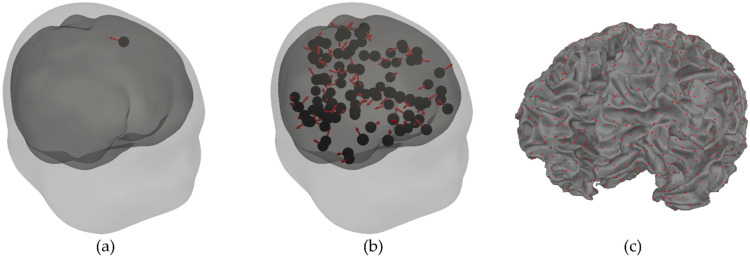
Examples of dipole locations and directions inside the brain. (**a**) Location and direction of one dipole for one sample time frame. The size of the red arrows does not represent the strength of the dipoles. (**b**) Locations and directions of 100 dipoles for simulated spontaneous brain activity for one sample time frame. (**c**) Example of the reconstructed brain surface (white matter) and all possible locations from which we chose the dipoles for the simulation (red dots).

For the sensor layouts listed above, we calculated the magnetic fields of current dipoles located inside the subject’s head. The forward model used a 3-layer BEM model [24]. The layers and the conductivities are as follows: scalp, 0.3 S/m; skull, 0.006 S/m; brain compartment, 0.3 S/m. For each layer, the outer BEM mesh consisted of 2562 vertices and 5120 triangles. All the geometries needed were reconstructed from the MRI image using the Freesurfer and MNE software package [25,26]. For one time sample, we simulated one dipole inside the brain. An example of one subject is shown in Figure 3a. Dipoles were chosen randomly for 100 time samples, each with a strength of q=100 nAm [27] and a random direction in the 3-dimensional Cartesian space. Each location was chosen randomly from a predefined source space, which was located on the outer mesh of the white matter (see Figure 3c). The simulations were performed for the head geometries (reconstructed MRI images) of 8 healthy subjects using the built-in functions of the software package MNE-Python [28].

We added two types of noise to each simulated magnetic field map (MFM). The first represents random ambient noise. The ambient noise ($B_{i}^{\mathrm{ambient}}\left(t\right)$) for one time frame (t) was calculated as:

(1) $$B_{i}^{\mathrm{ambient}}\left(t\right)=\sigma \xi_{i},$$

where σ represents the strength of the noise and $\xi_{i}$ is a Gaussian random number with a zero mean and a standard deviation equal to 1. The index i represents an individual sensor.

The other type of noise represents spontaneous brain activity, which originates inside the head. The noise for one sample was generated with 100 dipoles on the cortical mantle with randomly chosen locations and directions [20]. The strength of each “noisy” dipole was the same and was set with the parameter $q_{\mathrm{spont}}$. An example of 100 “noisy” dipoles for one subject and one time sample is shown in Figure 3b.

To evaluate how the noise affects the performance of different OPM-MEG layouts, we calculated the values of the signal-to-noise ratio (SNR) in units of dB [29]:

(2) $$\mathrm{SNR}=20\log_{10}\left(\frac{{\mathrm{RMS}}_{\mathrm{signal}}}{{\mathrm{RMS}}_{\mathrm{noise}}}\right),$$

where ${\mathrm{RMS}}_{\mathrm{noise}}$ is the root mean square of the generated noise and ${\mathrm{RMS}}_{\mathrm{signal}}$ is the root mean square of the data when we simulated the “expected” sources without the added noise.

To localize the source from the simulated magnetic fields, we performed an equivalent current dipole (ECD) fit for each time sample. We used the functions implemented in the MNE-Python software package [26,28,30]. For the forward model, we applied the same model as we used for generating the magnetic fields (the 3-layer BEM model). After obtaining the location and orientation for all time samples, we calculated the average distance between the simulated and fitted ECD ($d_{s,f}$).

As a last measure, we calculated the average relative error (RE) and the average correlation coefficient (CC) between the reconstructed MFM ($B^{r}$) and the simulated MFM ($B^{s}$) with added noise. For one time frame (t), these two values were calculated as:

(3) $$\mathrm{RE}=\frac{\bar{{\left(B^{s}-B^{r}\right)}^{2}}}{\bar{{\left(B^{r}\right)}^{2}}},$$

and

(4) $$\mathrm{CC}=\frac{\sum \left(B_{i}^{r}-\bar{B^{r}}\right)\left(B_{i}^{s}-\bar{B^{s}}\right)}{\sqrt{\sum {\left(B_{i}^{r}-\bar{B^{r}}\right)}^{2}}\sqrt{\sum {\left(B_{i}^{s}-\bar{B^{s}}\right)}^{2}}},$$

where B is an n-dimensional vector of magnetic fields and $B=\left(B_{1},B_{2},...,B_{n}\right)$; n is the number of sensors.

## 3. Results

**Figure 4:**
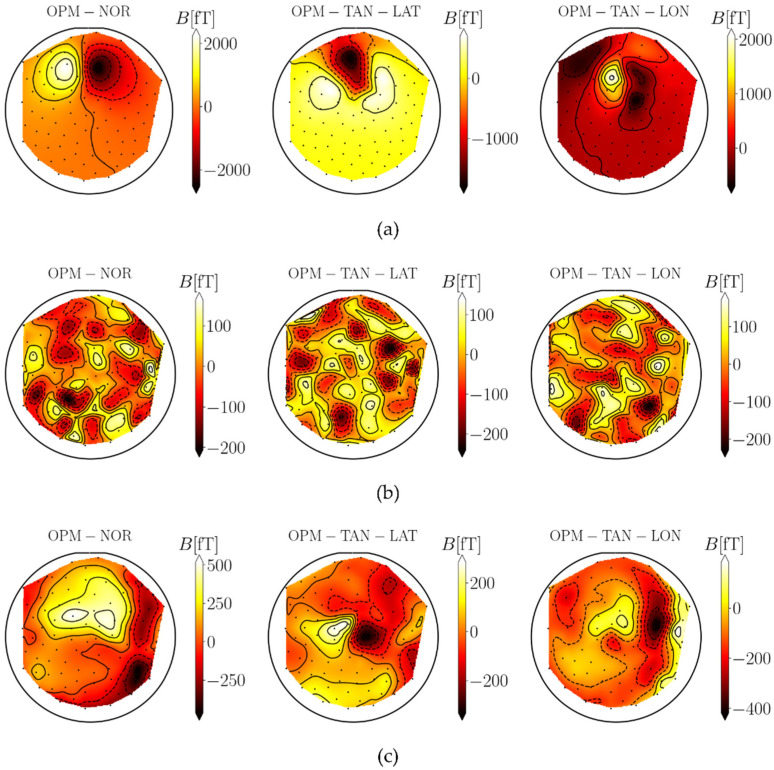
MFMs for one sample time frame for three cases: the simulated dipole without noise (**a**), simulated ambient noise (**b**), and spontaneous brain noise (**c**).

As the first result, we show the MFMs as contour plots and color-coded values of magnetic fields at the sensors’ locations. Brighter colors represent higher values of B. An example of one simulated dipole for all three OPM configurations on one subject for one time sample is shown in Figure 4a. MFMs for two different types of noise (ambient noise and spontaneous brain noise) for the same subject and for one time sample are shown in Figure 4b,c. We can see that for this example, the highest amplitude range for one dipole is for the OPM-NOR configuration. When we compare both noises, the ambient noise has higher heterogeneity, and we can see many local minima and maxima. The values for spontaneous brain noise are much more homogeneous.

As the next result, we present the average root mean square ($\bar{{\mathrm{RMS}}_{\mathrm{signal}}}$) values for all simulated MFMs without added noise. The $\bar{{\mathrm{RMS}}_{\mathrm{signal}}}$ for different OPM-MEG configurations are presented in Table 1 and those for different SQUID-MEG configurations are in Table 2. For both systems, we obtained the results in two steps. First, we calculated ${\mathrm{RMS}}_{\mathrm{signal}}$ for all 100 time samples for each subject. We then calculated the average $\bar{{\mathrm{RMS}}_{\mathrm{signal}}}$ and the standard deviation (SD) for all eight subjects. These two tables show that the $\bar{{\mathrm{RMS}}_{\mathrm{signal}}}$ was highest for the normal component for both the OPM- and SQUID-MEG. The OPM-MEG system has, on average, much higher ${\mathrm{RMS}}_{\mathrm{signal}}$ values.

**Figure 5:**
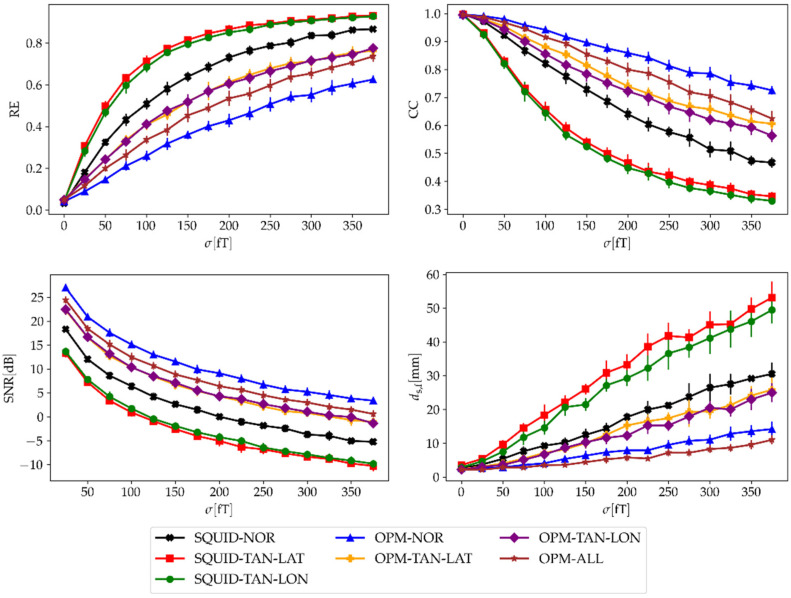
Evaluation parameters (RE, CC, SNR, and $d_{s,f}$) averaged over all 100 time samples and eight subjects in relation to the strength (σ) of the ambient noise for four different OPM-MEG configurations. The dipoles’ strength of spontaneous brain noise was $q_{\mathrm{spont}}=0$. Vertical lines represent the standard deviation between subjects.

As the next result, we changed the strength of both noises and checked how the evaluation parameters (RE, CC, SNR, and $d_{s,f}$) changed for both MEG systems. First, we changed the strength of the ambient noise from σ=0 to σ=375 fT. Each dipole’s strength of spontaneous brain noise was set to $q_{\mathrm{spont}}=0$. The results in Figure 5 show that the RE, CC, and SNR were the best when using the normal component only. The reason for this is that the largest generated fields are in the normal direction. However, the noise level is the same for all configurations. On the other hand, the localization accuracy ($d_{s,f}$) is the best when using all three components of the OPM-MEG system combined.

**Figure 6:**
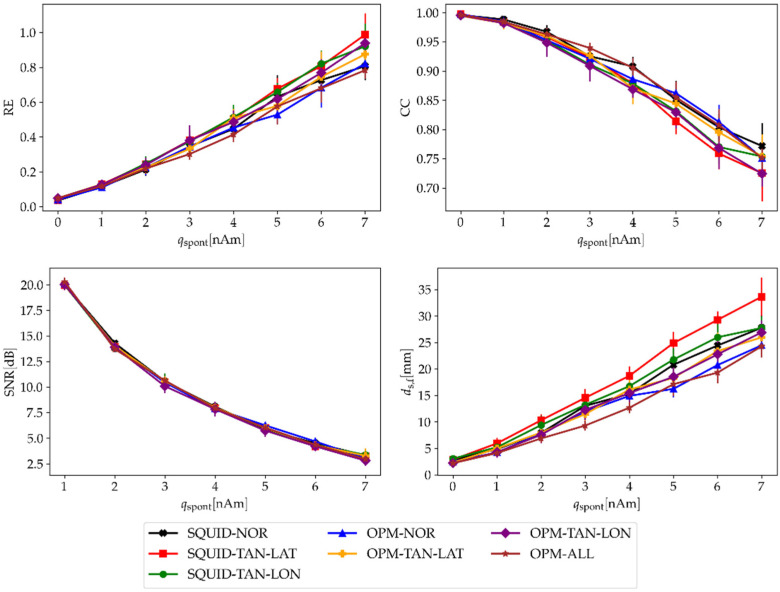
Evaluation parameters (RE, CC, SNR, and $d_{s,f}$) averaged over all 100 time samples and eight subjects in relation to the strength of the spontaneous brain noise ($q_{\mathrm{spont}}$) for four different OPM-MEG configurations. The strength of the ambient noise was σ=0. Vertical lines represent the standard deviation between subjects.

**Figure 7:**
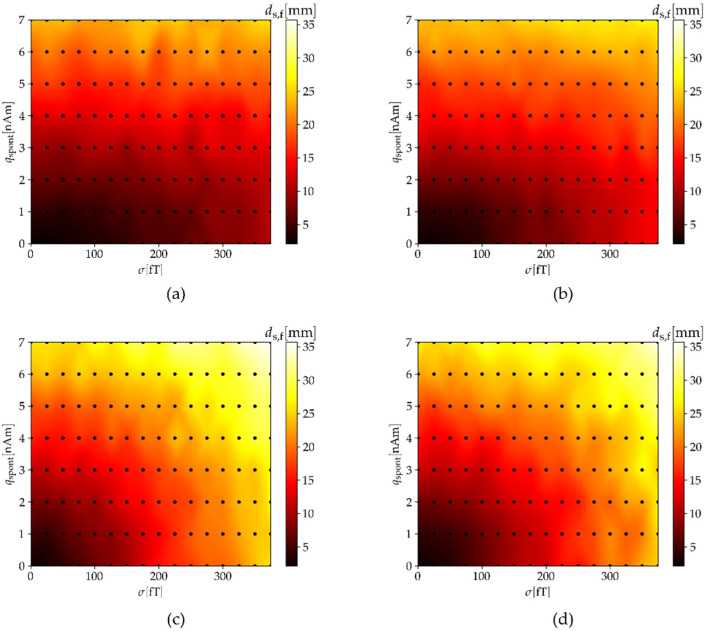
Average distance between the simulated and fitted dipoles ($d_{s,f}$) in relation to the strength of the spontaneous brain noise ($q_{\mathrm{spont}}$) and the strength (σ) of the ambient noise combined for four different OPM-MEG configurations: (**a**) OPM-ALL, (**b**) OPM-NOR, (**c**) OPM-TAN-LAT and (**d**) OPM-TAN-LON.

Next, we changed the strength of the spontaneous noise from $q_{\mathrm{spont}}=0$ to $q_{\mathrm{spont}}=7\;\mathrm{nAm}$ for both MEG systems. The strength of the ambient noise was σ=0. The results in Figure 6 show that the normal component does not have better results, as occurred in the previous case, because this component also has higher generated noise than the other two. If we look in detail, the localization accuracy ($d_{s,f}$) is also the best when using all three components. We expanded the results of $d_{s,f}$ by making calculations for different pairs of both noises $q_{\mathrm{spont}}$ and σ; the results are presented in Figure 7.

**Figure 8:**
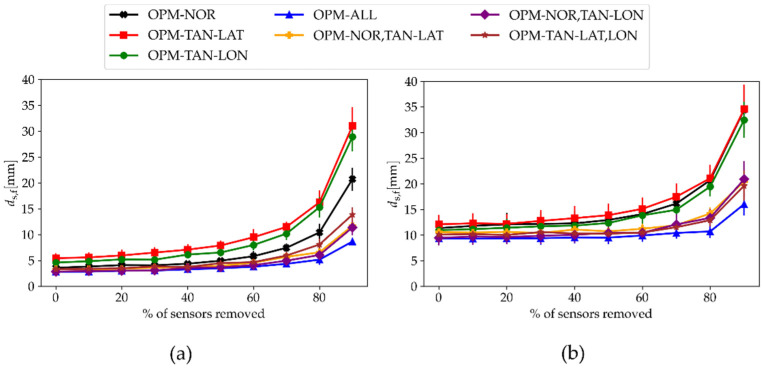
Distance between simulated and fitted dipoles ($d_{s,f}$) averaged over all 100 time samples and eight subjects in relation to the number of sensors for seven different OPM-MEG configurations. The strength of the ambient noise and the spontaneous brain noise was (**a**) σ=75 fT and $q_{\mathrm{spont}}=0$, and (**b**) σ=0 and $q_{\mathrm{spont}}=3\;\mathrm{nAm}$.

We checked how the number of sensors influenced the localization error $d_{s,f}$ when using seven different OPM-MEG configurations. In addition to the four OPM configurations presented in Figure 1, we also used three configurations in which we combined two orthogonal components: both OPM-NOR and OPM-TAN-LAT (OPM-NOR,TAN-LAT), both OPM-NOR and OPM-TAN-LON (OPM-NOR,TAN-LON), and both tangential components (OPM-TAN,LAT-LON). The simulation was performed for two cases: when we applied the ambient noise only (σ=75 fT, $q_{\mathrm{spont}}=0$) and when we applied the spontaneous brain noise only (σ=0, $q_{\mathrm{spont}}=3\;\mathrm{nAm}$). For each case, we gradually removed the sensors and performed the dipole fitting; the results are presented in Figure 8. Since the number of sensors for different subjects is not the same, we used the measure of the percentage of sensors we removed from the total number. We can see that for low sensor counts (a high percentage of sensors removed), multiple components yielded noticeably lowered the localization errors ($d_{s,f}$).

**Figure 9:**
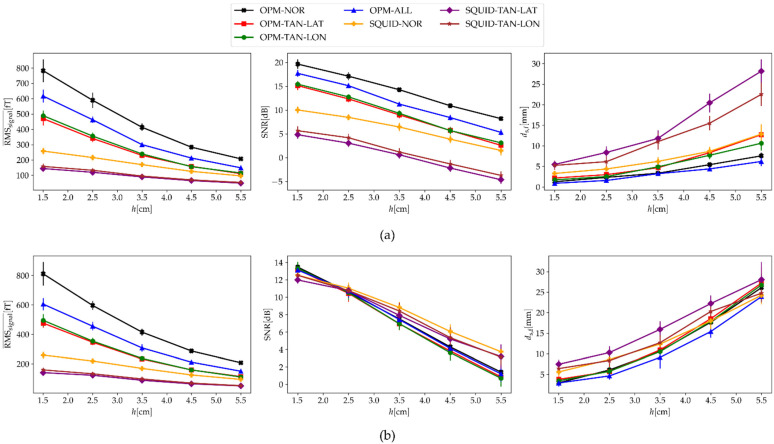
Calculated values of $\bar{{\mathrm{RMS}}_{\mathrm{signal}}}$, SNR, and $d_{s,f}$ in relation to the depth of the simulated dipoles (h). The strength of the ambient noise and the spontaneous brain noise was (**a**) σ=75 fT and $q_{\mathrm{spont}}=0$, and (**b**) σ=0 and $q_{\mathrm{spont}}=3\;\mathrm{nAm}$. Note that the value of h on the x-axis means, that the dipoles were chosen at a depth of h±dh; in our case, dh=0.5 cm.

Lastly, we tested how the depth of a simulated dipole affected the measured signal and the performance of a dipole fit for different sensor configurations (both OPM- and SQUID-MEG). For the previous simulations in this work, we picked 100 random dipoles from all possible locations (see Figure 3c), but for this case, we picked only dipoles that were at a certain depth (h). We defined the depth as the shortest Euclidean distance between the chosen dipole and the closest vertex of the outermost BEM mesh (the scalp layer). To evaluate the results, we calculated three measures: $\bar{{\mathrm{RMS}}_{\mathrm{signal}}}$, SNR, and $d_{s,f}$. The results are presented in Figure 9. Note that the value of h on the x-axis means that the dipoles were chosen at a depth of h±dh; in our case, dh=0.5 cm. For example, for the value h=1.5 cm, we chose 100 dipoles for which the shortest distance from the outer scalp layer was between 1.0 cm and 2.0 cm. The simulations were performed for two cases: when we added the ambient noise only (Figure 9a, σ=75 fT, $q_{\mathrm{spont}}=0$) and when we added the spontaneous brain noise only (Figure 9b, σ=0, $q_{\mathrm{spont}}=3\;\mathrm{nAm}$).

## 4. Discussion

The use of OPM sensors in MEG solves many disadvantages of the standard SQUID-MEG. Since these sensors do not require cooling with cryogenics, they can be placed directly on the scalp and thus allow the subject to move during measurements. This opens up a plethora of new applications for studying neural activity where the subject’s movement is required. In addition, the lower distance between the sensors and the head increases the strength of the measured signal, which, in theory, increases the SNR. This was already shown in a couple of previous works [6,7], and it was also confirmed by our results in Table 1 and Table 2, where we show that the OPM-MEG configurations have an $\bar{{\mathrm{RMS}}_{\mathrm{signal}}}$ around 2.5 higher than that of the SQUID-MEG configurations with magnetometers if we compare the same components from both MEG systems. The component with the highest magnetic fields is the normal component in both the OPM-MEG and in SQUID-MEG configurations; it had an $\bar{{\mathrm{RMS}}_{\mathrm{signal}}}$ almost two times higher compared with that of both tangential components. When we compared both tangential components, the component along the lines of longitude had, on average, a slightly higher $\bar{{\mathrm{RMS}}_{\mathrm{signal}}}$ than the component along the lines of latitude.

Next, we evaluated how noise affected different configurations of the measurement components of SQUID- and OPM-MEG. We added two different noises to each measurement component: ambient and spontaneous brain noise. The ambient noise represented sources from the outside environment; therefore, the amplitude of the noise was equal for both MEG systems and all components. This was simulated by adding a random field value to each channel. Spontaneous brain noise represented the subject’s spontaneous brain activity, i.e., sources inside the head. This noise was also used by Boto et al. [7], who simulated five noisy ECDs. In our work, we simulated 100 of them. This was simulated by distributing many dipoles randomly in the region of the cortex. Since it depends on the distance between sources and sensors, the noise level was higher for more closely positioned sensors. The effect of noise was checked by adding noise to the simulated MFMs and calculating the inverse problem (ECD fit), and by calculating the SNR. The goodness of fit was evaluated by calculating three measures: relative error (RE) and the correlation coefficient (CC), which were calculated between the noisy simulated MFMs and the fitted MFMs, and the distance between the simulated and fitted dipoles $d_{s,f}$. For solving the inverse problem, other methods could be used, such as minimum norm estimation, a music algorithm, or beamformers [31,32,33], the latter being the most popular in recent years. Although the beamformer approach has many advantages over more simple approaches, we consider that in our case, where we simulated data with single dipolar-like sources with added noise, the approach of fitting an ECD was the most appropriate. The results in Figure 5 show that adding ambient noise impacts each component and MEG system differently. OPM-MEG configurations yielded substantially lower localization errors compared with SQUID-MEG configurations (lower RE and $d_{s,f}$; higher CC and SNR). This is because the OPM sensors are closer to the sources inside the brain and therefore have a higher ${\mathrm{RMS}}_{\mathrm{signal}}$, but the ambient noise level is equal for all sensors. When we compared different components, the lowest impact was on the normal components of the magnetometers for both MEG systems. When we compared both tangential components, the results were very similar. For the OPM-MEG system, we additionally show that combining all three components lowered the value of $d_{s,f}$ even further. The finding that the radial component has a larger SNR than the tangential component on average was most likely the reason for the results observed in our recent study, where we transformed the data between OPM- and SQUID-MEG devices [13]. We obtained higher errors when transforming tangential components than when transforming the normal components of the OPM-MEG to the SQUID-MEG system, which measures the normal component of the magnetic field only.

Next, we added the spontaneous brain noise only (Figure 6). The SNRs of the SQUID-MEG configurations were not noticeably worse than the SNRs of OPM-MEG. This is because the OPM sensors are closer to the noisy sources inside the brain and therefore, the higher amplitude of the targeted brain source of interest does not increase the SNR. However, when we compare the parameter $d_{s,f}$, the results show that the OPM-MEG system is better. The same applies when we compare different OPM orientations, although the normal component has the largest field-generated ${\mathrm{RMS}}_{\mathrm{signal}}$; it has also higher values of ${\mathrm{RMS}}_{\mathrm{noise}}$. Therefore, the calculated SNR is very similar between configurations. This result is very interesting: although all configurations yielded almost identical SNRs, some configurations provided lower $d_{s,f}$ (OPM-NOR and OPM-ALL).

In real measurements, one deals with a combination of several noise sources. Here, we calculated the impact on the calculated parameter $d_{s,f}$ for a combination of spontaneous brain and ambient noise. We changed the parameters σ and $q_{\mathrm{spont}}$ for different sensing components of the OPM-MEG system (Figure 7). We observed that the distance $d_{s,f}$ began to increase the fastest for both tangential components (OPM-TAN-LAT and OPM-TAN-LON). The results are better and very similar for the OPM-MEG configurations OPM-NOR and OPM-ALL. This indicates that the addition of tangential components to the normal components of sensors does not significantly affect the dipole fitting results. Successful measurements with triaxial OPM sensors on subjects were performed in a recent study by Boto et al. [11]. In addition to the measurements, they performed a simulation for a triaxial OPM-MEG system with three head geometries (two children and one adult). They concluded that using triaxial sensors offers improved cortical coverage, especially in infants and children.

Adding additional tangential components also improves the performance of some methods which remove external disturbances, such as the signal space separation (SSS). In a study by Nurminen et al. [34], they added tangential SQUID sensors to a complete whole-head SQUID system. This increased the SSS shielding factor and reduced the reconstruction noise. The SSS method can also be used in OPM-MEG measurements. In [35], the authors hypothesized that using triaxial OPM sensors will result in the better performance of SSS and other spatial filter techniques.

Currently, commercial OPM-MEG systems with full-head coverage are rare. Although some companies offer viable solutions (Fieldline Inc., Boulder, CO, USA and Cerca Magnetics Limited, Nottingham, UK), many research groups buy the OPM sensors separately. Since the cost of these sensors is high, many use lower sensor counts. This approach reduces the initial investment and allows for future upgrades. In this work, we checked how lowering the number of sensors affected the distance between simulated and fitted dipoles ($d_{s,f}$) when using different components; additionally, we checked if combining multiple components lowered the error (Figure 8). Simulations were performed for two cases, where we used either ambient noise (Figure 8a) or spontaneous brain noise (Figure 8b). For the case with ambient noise and only one component, the worst results were obtained for the OPM-MEG configurations OPM-TAN-LAT and OPM-TAN-LON, and the best were for the OPM-NOR component. On the other hand, when we added spontaneous brain noise, the OPM-NOR component was not better compared with the other two, as all three components were inside the bounds of standard deviation. For a low percentage of sensors removed, the results were similar, as shown in Figure 5. For a high percentage of sensors removed, combining two or all three components significantly improved the result (lowered the $d_{s,f}$) compared with configurations with single components. The result was very interesting for the case with ambient noise: the configurations OPM-TAN-LAT and OPM-TAN-LON were very bad on their own, but combined (OPM-TAN-LAT, LON), they had a much lower $d_{s,f}$ than OPM-NOR. Combining all three components (OPM-ALL) yielded the best result (the lowest $d_{s,f}$). A similar finding was observed in [12], where they calculated the Frobenius norm of the forward field vector; the higher this norm, the lower the error in the beamformer projection. They showed that this value was higher for 50 triaxial sensors compared with 50 radial sensors. The main finding of the results shown in Figure 8 is that combining several components does not have a significant effect on the ECD fit localization result if one has many OPM sensors available. On the other hand, combining several components greatly improves the source localization results if one is limited by the sensor count (around 20 sensors or less). This can be utilized to greatly reduce the initial investment when building an OPM-MEG system. When we removed sensors, they were arranged equidistantly; it would be interesting to see how these results would change if the sensors were distributed optimally for a given ROI using an algorithm such as the one presented by Beltrachini et al. [36].

Another proposed advantage of OPMs compared with SQUIDs is the ability to measure signals from deep brain sources, such as the hippocampus [18,19]. In this simulation study, we tested this hypothesis and checked whether combining multiple OPM components had an advantage for deep brain sources. We simulated dipoles only at a specific depth (Figure 9); to the simulated MFMs, we added two types of noises (ambient noise and spontaneous brain noise) and performed ECD fitting. Even though the dipoles were randomly chosen for both noises, we obtained similar values for the $\bar{{\mathrm{RMS}}_{\mathrm{signal}}}$. The highest values of $\bar{{\mathrm{RMS}}_{\mathrm{signal}}}$ for all depths were calculated for the OPM-NOR configuration, followed by OPM-TAN-LAT and OPM-TAN-LON. The values for OPM-ALL were the mean values for all three OPM single components. The SQUID-MEG system had much lower values of $\bar{{\mathrm{RMS}}_{\mathrm{signal}}}$ compared with OPM-MEG; however, for the OPM-MEG configurations, they dropped more noticeably with an increasing h. For the ambient noise (Figure 9a) and for high values of h, the $d_{s,f}$ was lowest for the configuration OPM-ALL, followed very closely by OPM-NOR. The worst result was obtained by SQUID-TAN-LAT and SQUID-TAN-LON. This was expected, since SNR < 0. For the deepest simulated sources (h=5.5), SQUID-NOR had a similar $d_{s,f}$ to OPM-TAN-LAT. The calculated SNRs for the spontaneous brain noise (Figure 9b) were different from those for the ambient noise, and the values between the configurations are much more similar. This lack of a difference was observed for spontaneous brain noise (Figure 6) as well, since the amplitude ratio between brain noise and the brain source of interest stays the same irrespective of the distance between the sensor and the sources. For low values of h, the OPM configurations had slightly higher values of SNR; on the contrary, for high values of h, SQUIDs had a higher SNR. For this noise, OPM-ALL also had the lowest $d_{s,f}$, but this was significant only for the middle values of h. For low values of h, the OPM-MEG configurations had lower values of $d_{s,f}$ than SQUID-MEG, but for high values of h, SQUID-MEG had slightly lower values of $d_{s,f}$, except for OPM-ALL and SQUID-TAN-LON

In this simulation study, we assumed that both types of sensors had equal sensitivities. An actual SQUID MEG system has a typical sensitivity of $3\;\mathrm{fT}/\sqrt{\mathrm{Hz}}$ [37], and the OPMs produced by QuSpin (https://quspin.com/products-qzfm/, accessed on 6 April 2022) have sensitivities of $7–10\;\mathrm{fT}/\sqrt{\mathrm{Hz}}$ and $15\;\mathrm{fT}/\sqrt{\mathrm{Hz}}$ for operating in dual and triple axis mode, respectively. This intrinsic sensor noise could be considered as an additional uncorrelated contribution to the random ambient noise. Consequently, the variance ($\sigma^{2}$) of the combined ambient and intrinsic noises can be determined by the sum of the variances of both noises. Therefore, one can conclude that for ambient noise levels above 30 fT (σ>30 fT), the addition of the intrinsic noise would not significantly change the outcome, which was confirmed by our results displayed in Figure 5. The same conclusion could be drawn from the results in Figure 8a and Figure 9a, where σ=75 fT was considered. For deeper brain sources, the relative distances between the sensors and sources are not so much in favor of OPMs in comparison with SQUIDs anymore. The gain in the $\bar{{\mathrm{RMS}}_{\mathrm{signal}}}$ is only about a factor of 2. However, one can estimate, through simple calculus, that in the presence of combined intrinsic and ambient random noises, the SQUIDs would outperform the OPMs in terms of a better SNR only for very low levels of random ambient noise (σ<8 fT).

## 5. Conclusions

In this study, we performed a numerical simulation to test the performance of different measurement components of both SQUID- and OPM-MEG systems. The sensors in the OPM-MEG system were closer to the source than those in the SQUID-MEG system; therefore, they measured higher magnetic fields. For this reason, the SNR and the source localization accuracy was higher for the OPM-MEG system when we introduced ambient noise. For both systems, the normal direction of the sensors yielded the lowest source localization error compared with the other two orthogonal directions. When we combined all three directions, the error was even lower. Since SQUID-MEG rarely has access to all three directions of magnetic fields, tri-axial OPM-MEG has a clear advantage over SQUID-MEG.

When introducing the spontaneous brain noise, the SNR for all the sensor configurations was almost the same, within the range of error. Nevertheless, for OPM, the normal components and all components combined had slightly lower localization errors. We showed that for low sensor counts, which are relevant, given the currently high costs of OPMs, combining multiple components significantly improves the source localization results compared with single-component OPMs.

Lastly, we performed simulations for different dipole depths. When we added the ambient noise, the OPM-MEG performed much better than the SQUID-MEG configurations, while this was not the case for spontaneous brain noise. Combining all three components improved the localization results for both types of noise. This finding would be worth verifying in future work with actual measurements of the signals from deep brain sources.

## Figures and Tables

**Table 1:** The average ${\mathrm{RMS}}_{\mathrm{signal}}$ of the simulated MFM without noise for four different OPM-MEG configurations.

Configuration	OPM-ALL	OPM-NOR	OPM-TAN-LAT	OPM-TAN-LON
$\bar{{\mathrm{RMS}}_{\mathrm{signal}}}\pm \mathrm{SD}$	375 fT ± 30 fT	459 fT ± 37 fT	277 fT ± 30 fT	284 fT ± 26 fT

**Table 2:** The average ${\mathrm{RMS}}_{\mathrm{signal}}$ of the simulated MFM without noise for four different SQUID-MEG configurations.

Configuration	SQUID-AXI-GRAD	SQUID-NOR	SQUID-TAN-LAT	SQUID-TAN-LON
$\bar{{\mathrm{RMS}}_{\mathrm{signal}}}\pm \mathrm{SD}$	132 fT ± 13 fT	189 fT ± 9 fT	100 fT ± 14 fT	109 fT ± 10 fT

## Data Availability

The data presented in this study are available on request from the corresponding author. The data are not publicly available due to the nonstandard file formats.
